# Gene-metabolite networks associated with impediment of bone fracture repair in spaceflight

**DOI:** 10.1016/j.csbj.2021.05.050

**Published:** 2021-06-08

**Authors:** Nabarun Chakraborty, Ariane Zamarioli, Aarti Gautam, Ross Campbell, Stephen K Mendenhall, Paul J. Childress, George Dimitrov, Bintu Sowe, Aamir Tucker, Liming Zhao, Rasha Hammamieh, Melissa A. Kacena

**Affiliations:** a Medical Readiness Systems Biology, CMPN, WRAIR, Silver Spring, MD, USA; b Department of Orthopaedic Surgery, Indiana University School of Medicine, Indianapolis, IN, USA; c Department of Orthopaedics and Anaesthesiology, Ribeirão Preto Medical School, SP, Brazil; d Geneva Foundation, Medical Readiness Systems Biology, CMPN, WRAIR, Silver Spring, MD, USA; e ORISE, Medical Readiness Systems Biology, CMPN, WRAIR, Silver Spring, MD, USA; f Department of Neurological Surgery, Indiana University School of Medicine, Indianapolis, IN, USA; g Richard L. Roudebush VA Medical Center, Indianapolis, IN, USA

**Keywords:** Genomics, Gene-metabolite network, Metabolomics, Bone, Bone defect, Mouse model, Space

## Abstract

Adverse effects of spaceflight on musculoskeletal health increase the risk of bone injury and impairment of fracture healing. Its yet elusive molecular comprehension warrants immediate attention, since space travel is becoming more frequent. Here we examined the effects of spaceflight on bone fracture healing using a 2 mm femoral segmental bone defect (SBD) model. Forty, 9-week-old, male C57BL/6J mice were randomized into 4 groups: 1) Sham surgery on Ground (G-Sham); 2) Sham surgery housed in Spaceflight (FLT-Sham); 3) SBD surgery on Ground (G-Surgery); and 4) SBD surgery housed in Spaceflight (FLT-Surgery). Surgery procedures occurred 4 days prior to launch; post-launch, the spaceflight mice were house in the rodent habitats on the International Space Station (ISS) for approximately 4 weeks before euthanasia. Mice remaining on the Earth were subjected to identical housing and experimental conditions. The right femur from half of the spaceflight and ground groups was investigated by micro-computed tomography (µCT). In the remaining mice, the callus regions from surgery groups and corresponding femoral segments in sham mice were probed by global transcriptomic and metabolomic assays. µCT confirmed escalated bone loss in FLT-Sham compared to G-Sham mice. Comparing to their respective on-ground counterparts, the morbidity gene-network signal was inhibited in sham spaceflight mice but activated in the spaceflight callus. µCT analyses of spaceflight callus revealed increased trabecular spacing and decreased trabecular connectivity. Activated apoptotic signals in spaceflight callus were synchronized with inhibited cell migration signals that potentially hindered the wound site to recruit growth factors. A major pro-apoptotic and anti-migration gene network, namely the RANK-NFκB axis, emerged as the central node in spaceflight callus. Concluding, spaceflight suppressed a unique biomolecular mechanism in callus tissue to facilitate a failed regeneration, which merits a customized intervention strategy.

## 1 Introduction

There is a legitimate concern that the compromised musculoskeletal physiology in spaceflight will place astronauts at a high risk for bone fracture [1], [2], [3], [4]. The musculoskeletal disuse driven by chronic unloading is probably the major contributing factor for the bone loss in spaceflight [5], [6]. Moreover, compromised musculoskeletal health could be causally related to the “polytraumatic milieu” of spaceflight, which embodies complex relationships among weightlessness, radiation, and various disrupted biological functions, including circadian rhythm, immune functions, and metabolism [7]. It is likely that this polytraumatic milieu in spaceflight could also be detrimental to fracture healing by diminishing the chances of recovery. This idea is supported by our previous study showing that spaceflight potentially damages several key regenerative mechanisms by suppressing bioenergetics, triggering immunodeficiency, and inhibiting growth factors *in vitro* [7], [8], [9].

The acute response of bone fracture healing is an energy expensive process that fosters the recruitment of growth factors and cytokines to initiate recovery [10]. Spaceflight can cause energy deprivation [9] and potentially impedes recruiting healing-critical agents such as growth factors at the wound site [8]; therefore, it would be difficult to sustain a self-healing process in spaceflight, and a countermeasure would be necessary. Nevertheless, identifying a spaceflight-specific therapeutic target is a major challenge, since a holistic characterization of its healing cascade is difficult to comprehend due to limited availability of spaceflight data [11]. Past reports estimated that the astronauts’ femoral neck strength decreased at a rate of 5.0% per month in spaceflight [12]. Additional reports predicted that the spaceflight-induced cortical and trabecular bone mass loss could have long-term, adverse consequences to overall bone integrity [13], [14]; particularly when the astronauts return to Earth or land on a reduced gravity planetary surface, such as Mars or the Moon [15]. The positive correlation between spaceflight duration and compromised musculoskeletal health was underscored by a predictive bone fracture model [16].

Fracture non-union remains an important orthopedic complication. A complex and well-orchestrated series of physiological events are necessary for successful bone healing and regeneration [10], [17]. Overall, these events are broadly classified into two overlapping phases, namely the metabolic phase and biological phase. The metabolic phase begins with anabolic processes that includes the initiation of skeletal and vascular tissues. At the transition stage from the anabolic to catabolic activities, the bone secondary structures begin to form. Eventually, the catabolic activities predominate as bone structure regains its pre-injury state [10]. This entire episode is supported by three major biological phases that longitudinally advance from the inflammatory phase, to the endochondral bone formation phase, to the bone remodeling phase. Sub-optimal performance of these metabolic and/or biological phases can result in impaired bone regeneration.

Current reports indicate that approximately 12.5% of the femoral shaft fractures result in non-union [18], [19]. Clinical management of traumatic or infection induced segmental bone defect (SBD) injuries, typically involves bone grafting with a frame or use of cement spacers as commonly referred to as the Masquelet technique [20] followed by bone grafting (a 2-stage procedure). In some cases, at the time of surgery, surgeons apply a collagen membrane impregnated with bone morphogenetic protein (BMP) to the SBD site to improve bone healing. On the other hand, clinical management of SBDs following tumor resection typically require bulk allograft or similar “scaffolding” materials for successful bone repair. Many times orthopedic surgeons use hollow rods to stabilize the fracture site. Most patients with SBD injuries will undergo prolonged rehabilitation and limited weightbearing/bedrest following surgery [21], [22]. Prolonged skeletal unloading often causes osteopenia to further complicate the healing trajectory [23]. Clearly, SBDs are anatomically and medically complex injuries that are remarkably difficult to treat, and prolonged disuse of limbs, such is the condition in spaceflight and the bedridden patient, further complicates recovery [22].

Characterization of the interconnectedness between the spaceflight induced prolonged disuse and bone fracture is critical to design precise osteoinductive interventions for space travelers. The present spaceflight study used a critical sized, 2 mm SBD model, which does not typically heal on the ground without additional therapeutic intervention, such as treatment with BMP-2 or other bone healing drugs. We selected this SBD model as compared to a closed fracture model for multiple reasons, such as: 1) SBD’s standardized fracture pattern (e.g. reduced comminution and misalignment), fracture location, and the reduction of associated complications compared to the use of the closed Einhorn fracture model [24]; and 2) the potentially reduced soft tissue injury in SBD model compared to the closed Einhorn fracture model (e.g. crush injury to the quadriceps) [25]. In addition, SBD model is an ideal platform to investigate the efficacy of bone healing drugs [26], [27].

We hypothesize that this femoral SBD mouse model [28], when subjected to spaceflight, will trigger a differential fracture healing trajectory compared to that in the mice with SBD on ground. In concert, the pan-omics landscape will likely be altered via a unique fashion because of the cumulative impact of bone injury and spaceflight induced stress. To meet this objective, our Rodent Research 4 (RR4) project (https://www.nasa.gov/ames/research/space-biosciences/rodent-research-4-spacex-10) investigated the impact of spaceflight on *in vivo* SBD repair. C57BL/6J mice with and without SBD surgery were group-housed in the International Space Station (ISS) for 24–28 days. Age-matched mice with and without SBDs were housed on the Earth at Kennedy Space Center (KSC) inside identical spaceflight hardware maintained in identical conditions including diet, temperature, light cycle, humidity, etc. Our previous µCT results from investigating this RR4 cohort [3] revealed that spaceflight (without surgery) and surgery (on ground or in spaceflight), all resulted in a loss of trabecular bone in the ipsilateral tibia. However, the mode of consequences of all three stressful scenarios were apparently different from each other. In other words, the trabecular bone loss in the ipsilateral tibia in spaceflight mice subjected to SBD surgery was not entirely an accumulative effect of the surgery on ground and the effects of spaceflight on sham mice. Our additional study to probe the adaptation process of the skeleton to spaceflight [4] underscored the necessity in examining the local wound site to better characterize the influence of spaceflight on the SBD repair processes at a single snapshot in time (∼4 weeks post-surgery). Here we present a systems integration of global transcriptomic and metabolomic assays tied with µCT readouts of the callus to find the underlying molecular mechanisms of bone fracture healing with and without the spaceflight induced unloading. This bone related outcome is also presented in the context of past studies on the quadriceps, where multi-omics investigation revealed significant energy deprivation in weight-bearing muscles of sham mice in the spaceflight [9]. Finally, these analyses will serve as key baseline data for future comparisons to spaceflight SBD mice treated with BMP-2 or other bone healing agents.

## 2 Materials and methods

### 2.1 Animals

All experiments were performed in accordance with the NIH Guide for the Care and Use of Laboratory Animals and followed experimental protocols approved by the NASA Animal Care and Use Committees, #FLT-15-101/NAS-15-105 and Animal Care and Use Review Office (ACURO) of U.S. Army Medical Research and Development Command (USAMRDC).

**Fig. 1:**
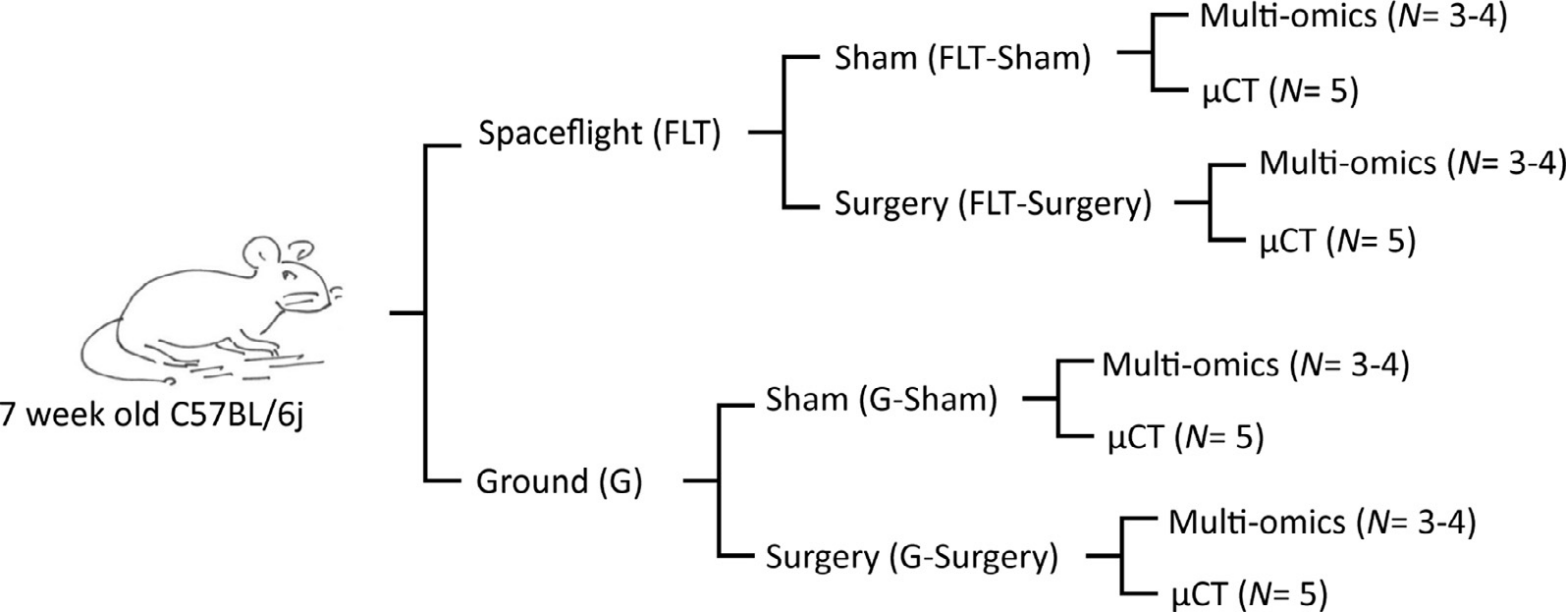
The study design. Seven weeks old male C57BL/6J mice were randomly divided by cages into two groups, one was stipulated to be launched to space (FLT) and the other was housed on Earth as controls on Ground (G). Within these two groups, two subgroups were generated; one subgroup underwent Surgery and the other served as unoperated Shams. Each subgroup was further divided equally for assay purposes. Approximately half were used for multi-omics assays (n = 3–4) and half were used for micro-computed tomography (µCT) analysis (n = 4–5).

Seven-week-old male C57BL/6J mice were purchased from Jackson Laboratories (Bar Harbor, ME). Fig. 1 describes the flow diagram of the animal handling protocol. The mice were cage-mated from weaning in N40 mouse cages in groups of 15 mice (Ancare, Bellmore, NY). The mice were then acclimated to their new environment and experimental housing conditions as previously described [28], [29], [30], [31], [32]. Briefly, mice were housed in a 12-hour light/dark cycle condition and at temperature of 24–25 °C. The mice were placed in N40 cages with a raised wire floor (3 openings/inch, Ancare), fed NASA Nutrient-upgraded Rodent Food Bar (NuRFB), and provided modified lixit water bottles to simulate spaceflight-like hardware/food. Mice were ear punched for identification and weighed biweekly to evaluate their adaptation to the special water lixit and NuRFB.

### 2.2 Spaceflight and ground protocol for animal handling

Fig. S1 depicts the workflow diagram for the entire protocol, which was designed around the stipulated time for the spaceflight launch. Four days prior to launch, after an acclimation period of 10 days, the mouse cages were randomized into 4 groups (Fig. 1): 1) Sham surgery on Ground (G-Sham); 2) Sham surgery housed in Spaceflight (FLT-Sham); 3) SBD surgery on Ground (G-Surgery); and 4) SBD surgery housed in Spaceflight (FLT-Surgery). The surgical procedure was carried out as previously described [28], [29], [32]. Briefly, mice were anesthetized with Ketamine-Xylazine (125–20 mg/kg), and the right hindleg was shaved from 1 cm distal to the knee joint to midline. The leg was then prepped with ethanol and Betadine three times each in an alternating fashion to ensure limb sterility. After the anesthetic plane was confirmed, a one cm incision was then made over the right femoral midshaft extending to the knee joint. The knee was flexed, and a 27-gauge hypodermic needle was inserted into the distal end of the femur and passed in a retrograde fashion into the intramedullary canal. A sterile Dremel rotary cutting tool (Dremel Inc. Racine, WI) was then used to remove a 2 mm segment from the midpoint of the femoral diaphysis. Next, a previously characterized, custom made, 2 mm poly(propylene fumarte)/tricalcium phosphate graft was inserted at the defect site to maintain defect size [33], [34], [35], [36], [37], [38], [39]. The previously placed needle was advanced through the graft and proximal femoral intramedullary canal through the greater trochanter. The needle tip was then bent back on itself and the needle pulled anterograde to stabilize and align the femoral defect. The needle was then cut at the distal end of the femur and a saline-soaked collagen sponge (RCM6 Resorbable Collagen Membrane, ACE, Brockton, MA) was wrapped around the scaffold and sutured into place. The muscle was closed with simple Vicryl suture and the skin was closed using wound clips (7 mm, Braintree Scientific, Braintree, MA). Once the mice recovered from anesthesia, they were provided with buprenorphine (0.05 mg/kg) for analgesia, and then were returned to their original cages with K3392 Rest Stops (Bio-Serve, Flemington, NJ) for the first 2 days to assist with recovery. Of note, surgical complications were observed in less than 5% of the mice. After the 2-day recovery period, the 10 healthiest mice per group (as determined by NASA veterinarians in collaboration with authors MAK and PJC by visual inspection of surgery sites, the overall activity levels, and condition of the mice) were transferred into spaceflight housing 2 days prior to launch. The FLT-Sham and FLT-Surgery mice were housed in NASA Rodent Transporters while in the SpaceX Dragon capsule, and on February 19, 2017, these mice were launched into space via Space X-10 vehicle operated under commercial resupply services (CRS)-10 mission [40]. After 5 days, they were moved to International Space Station’s (ISS) NASA rodent research Habitat (https://www.nasa.gov/ames/research/space-biosciences/rodent-research-hardware), where they lived for the next 24–28 days until euthanasia. It should be noted that upon transfer of mice to the Habitat hardware on the ISS (and on the ground at KSC), the 10 group-housed Sham or Surgery mice were randomly subdivided into 2 groups of 5 mice because the Habitat holds a maximum of 5 mice per side (2 sides/Habitat). The G-Sham and G-Surgery cohorts were housed in identical spaceflight hardware and treated as identically as possible; 5 days asynchronous to spaceflight cohorts in order to best simulate spaceflight conditions. Importantly, weekly orders of mice were delivered from Jackson Laboratories to ensure properly aged mice for possible delays in launch. Therefore asynchronous ground controls were ∼2 days younger in age than flight mice, which is negligible in terms of bone status/growth in 9- and 13- week-old mice (the approximate age and the time of surgery and euthanasia). To note, this manuscript represents 20 spaceflight mice, which was a part of a total of 40 mice that were sent to the ISS during this mission. The other 20 mice also had surgery but were treated with BMP-2 and a novel bone healing agent, thrombopoietin. Those data are still being processed and will be described in future reports.

### 2.3 Tissue sample collection

Mice were euthanized at 24–28 days post-launch when mice were approximately 13 weeks of age. The extended time range was due to the significant time it takes to accomplish tasks in spaceflight. For these studies, the astronauts were able to euthanize and collect samples from a maximum of 8 mice per day. Euthanasia was performed by injection of ketamine/xylazine (150/45 mg/kg) followed by closed chest cardiac puncture with blood withdrawal and cervical dislocation. Each group of 10 mice was subdivided into two groups of 5 mice. For 5 mice/group, the right hindlimb was removed at the hip and stored in 10% neutral buffered formalin (NBF) at 4 °C until they were returned to Indiana University School of Medicine (IUSM) approximately 2 weeks after euthanasia. After arrival at IUSM, the right hindlimb samples were washed with ice cold phosphate-buffered saline (PBS), transferred to ice cold 70% ethanol and stored at 4 °C until being imaged by micro-computed tomography (µCT). The remaining mouse carcass was stored in aluminum foil in NASA’s cold stowage, which is at or below −80 °C on the ISS until transfer back to cold storage on Earth at −80 °C. For the other one-half of mice in each group, the whole carcass was left intact and stored in aluminum foil at or below −80 °C. The carcasses remained at −80 °C or lower until they arrived at the Walter Reed Army Institute of Research (erstwhile, US Army Center for Environmental Health Research or USACEHR) at Fort Detrick, MD approximately 2 weeks after euthanasia.

Following a well-practiced protocol, the mice were then partially thawed and tissue dissection for each carcass was completed in approximately 45 min. After dissection, individual tissues were snap frozen in liquid nitrogen and stored at −80 °C until processing.

Most relevant to the present study, the proximal and distal 2 mm of the femur of surgery mice (both FLT and G) were removed and the remainder of the midshaft was considered the callus regions. The same region of the sham femurs (both FLT and G) were saved as controls. Therefore, there were a maximum 5 mice/group allotted for both µCT analyses and multi-omics assays.

### 2.4 Micro-computed tomography

**Fig. 2:**
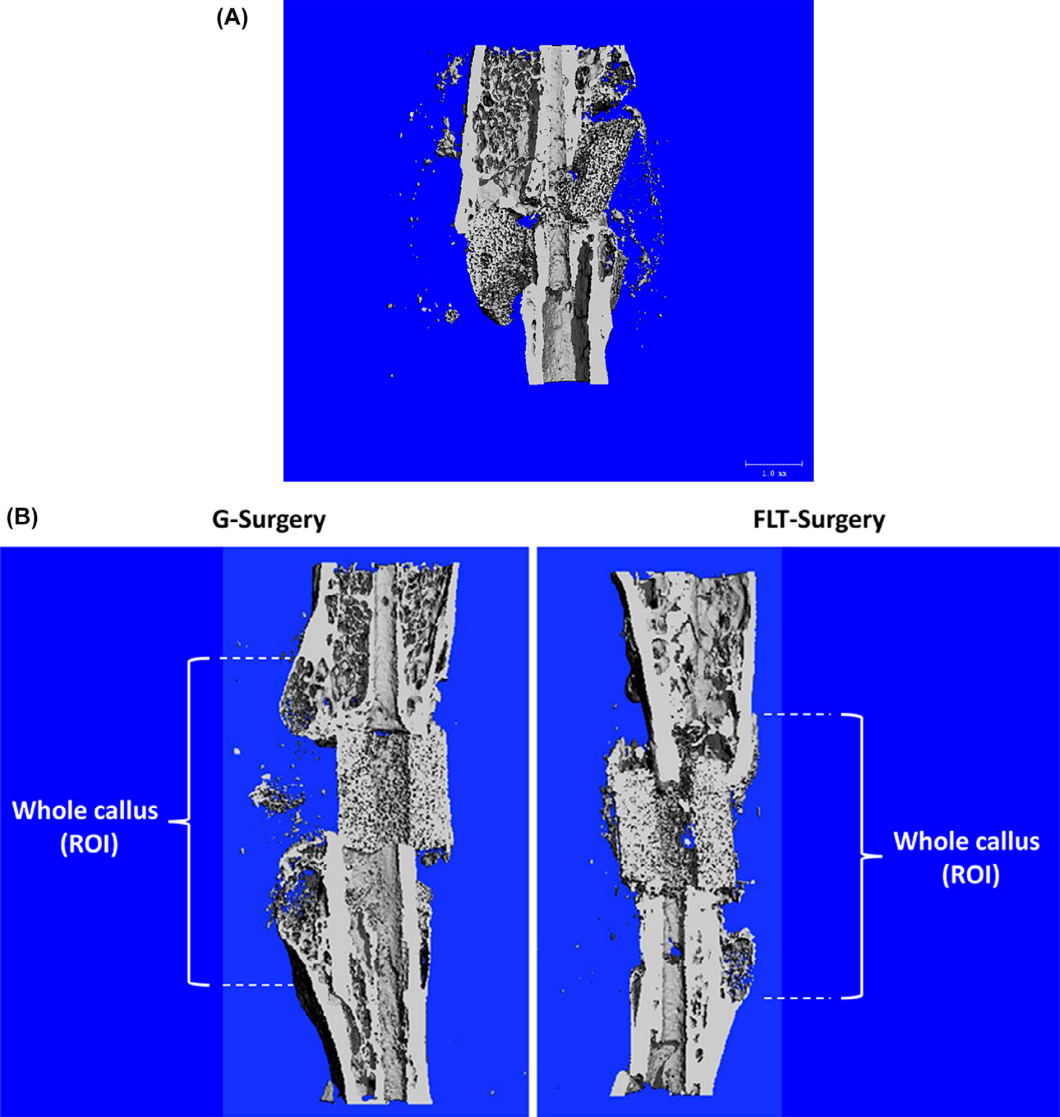
Microcomputed images of femurs containing 2 mm SBD approximately 4 weeks after surgery. (A) Microcomputed image showed hardware failures only observed in G-Surgery mice. On ground, the SBD mouse typically becomes ambulatory immediately after recovery from anesthesia. This application of pressure to the scaffold can it break over time if healing does not occur. Unloading in spaceflight potentially allowed the scaffold to remain intact for a longer period of time. No FLT-Surgery picture is added, since this incidence was ground specific. (B) The images showed that complete bone bridging was not observed in either the G-Surgery or FLT-Surgery mice. Without any therapeutic intervention, this size of SBD does not typically heal on ground, and the present study showed that prolonged disuse of limbs in spaceflight also cannot facilitate healing of the 2 mm SBD. The region of interest or the callus area is shown.

Prior to µCT analysis, all callus segments (or equivalent femora sections of sham mice) were briefly thawed and the surrounding soft tissue removed. Surgical pins were carefully removed at this time. Each femur was then fixed for 72 h in 10% NBF, washed with PBS, and stored in 70% ethanol at 4 °C. All femora were imaged using a desktop SCANCO µCT35 imaging system (SCANCO Medical, Bruttisellen, Switzerland). All scans were obtained at 55 kV using a 12 µm voxel size. A 3D analysis was performed taking the entire callus area as the region of interest (see Fig. 2B) in which the following morphometric parameters were measured: (1) tissue volume (TV), in mm^3^: the total callus volume; (2) bone volume (BV)/TV, in %: the woven bone fraction, which was expressed as a percentage of the callus volume; (3) connectivity density (Conn.D), in 1/mm^3^: the connectivity density normalized by TV; (4) structure model index (SMI), where 0 stands for parallel plates and 3 for cylindrical rods; (5) tissue mineral density (TMD), in 1/cm^3^: mean callus density; (6) bone surface (BS), in mm^2^: bone surface area; (7) BS/BV, in 1/mm: relative bone surface; (8) trabecular number (Tb.N), in 1/mm; (9) trabecular thickness (Tb.Th), in mm; and (10) trabecular separation (Tb.Sp), in mm. The callus area or region of interest was manually drawn using the SCANCO software for each specimen by one reader who was blinded to groupings. The cortical bone and the scaffolds were isolated and were not included in the analyses. Further, it should be noted that the nomenclature used is in accordance to the American Society for Bone and Mineral Research [41]; however, these parameters are for standard bone parameters and while not specific for fracture healing, they are commonly adapted for assessing callus quantity and quality. Additionally, fracture healing was assessed using the modified Radiographic Union Score for Tibial fractures method (RUST) [42], [43]. Using this method, the medial, lateral, anterior, and posterior cortices on anteroposterior and lateral µCT images were scored. Each cortex was given a score of 1 (no bridging), 2 (partial bridging), or 3 (complete bridging). The total score is equal to the sum of the scores from all 4 cortices ranging from 4 (not healed) to 12 (maximally healed). All images were scored by 3 blinded readers and the average scores were reported.

### 2.5 mRNA extraction

Extraction of RNA from frozen tissues was carried out following an established protocol [44]. Frozen callus segments (or equivalent femora sections of sham mice) were cryogenically grounded using Cryomill (Retsh GmbH, Germany) and the amorphous materials were divided into two portions; one for transcriptomic and the other for metabolomic assays. TRIzol reagent (Invitrogen, Thermo Fisher Scientific, Wilmington, MA) was added to the portions marked for transcriptomic assays and the specimens were homogenized using a Precellys Evolution Homogenizer and Cryolys Evolution (Bertin Technologies SAS, France). Invitrogen TRIzol RNA extraction methods and the miRNeasy mini kit total RNA purification procedures (QIAGEN Inc., Germantown, MD) were followed to extract the mRNA. The quantity and quality of RNA was then determined using the NanoDrop 2000 spectrometer (Thermo Fisher, Wilmington, DE) and the Agilent 2100 Bioanalyzer (Agilient Technologies, Inc., Santa Clara, CA), respectively. The purified RNA samples were stored at −80 °C for future use.

### 2.6 Whole genome expression analysis

Dual dye microarray was performed using the SurePrint G3 mouse GE 8 × 60 k microarray kit (Agilent Technologies, Inc., CA, USA) following a well-established protocol [44], [45]. This whole mouse genome microarray slides contains 41,000 mouse transcripts. Briefly, purified RNA was labeled with Cy-5 dyes. The reference RNA (Agilent, CA) was labeled with Cy-3 dyes. The samples were hybridized and incubated for 17 h at 55 °C. After overnight incubation, the slides underwent a series of washes. The slides were imaged using an Agilent DNA microarray scanner and the features were extracted using the default settings of the manufacturer software (Feature Extraction software v.10.7, Agilent, CA).

Data filtration and statistical analysis was performed on GeneSpring v10.1 (Agilent Technologies, Inc., CA). Each chip was subjected to intra-chip normalization using the locally weighted scatterplot smoothing method or LOWESS method [46]. To find the differentially expressed probes between FLT-Sham and G-Sham, the cut-off was set at a fold change ≥ |2| and a moderate *t*-test *p* ≤ 0.01 was used. The same cut-off was set to find the differentially expressed probes between FLT-Surgery and G-Surgery. Subsequently, the differentially expressed probes were mapped to the Agilent provided gene database to identify differentially expressed genes (DEGs). The microarray data was submitted to the Gene Expression Omnibus (GEO). This can be searched using the Accession number: GSE161618.

### 2.7 Real-time PCR validation of microarray results

A group of genes was selected for the validation assay based on their relevance to this study (pertinent comments based on literature survey were noted in Table 3), their high expression values (Fold change > 2) and their association with the NFκB network (genes enriching this particular network were underlined in Table 3). This assay was performed following a protocol described elsewhere [47]. Briefly, from 100 ng of total RNA, a cDNA library was prepared using the RT^2^ First Strand Kit (Applied Biosystems, Foster City, CA). Real-time PCR was performed on QuantStudio™ 7 Flex System (Applied Biosystems) equipped with QuantStudio Real-Time PCR Software using primers and PCR Master Mix, both purchased from Applied Biosystems. The samples were run in triplicates. Table S1 lists the primers used for this study. Glyceraldehyde-3-Phosphate Dehydrogenase (GAPDH) was used as a control gene. Thermal cycling conditions were 2 min at 50 °C and 10 min at 95 °C followed by 40 cycles at 95 °C for 15 sec and at 60 °C for 1 min. The ΔΔCT method was used for data analysis [48].

### 2.8 Metabolomics assay

The second aliquot of bone tissue homogenates was used for untargeted metabolomics assay using Quadruple Time-of-Flight mass spectrometer (Q-TOF MS) (Waters, Inc., Milford, MA) following a previously documented protocol [49], [50], [51]. Briefly, 50 mg of the homogenized tissue was dissolved in chilled extraction buffer (50% methanol in water) and the protein containing phase was removed as the proteins were precipitated by addition of equal volumes of acetonitrile. Five microliters of the protein-free sample was injected onto a reverse-phase 50 × 2.1 mm Acquity 1.7-μm C18 column (Waters Corp) using an Acquity Ultra Performance Liquid Chromatography (UPLC) system (Waters) with a gradient mobile phase consisting of 2% acetonitrile in water mixed with 0.1% formic acid (Solvent A) and 2% water in acetonitrile mixed with 0.1% formic acid (Solvent B); and the column was resolved for 10 min at a flow rate of 0.5 mL/min. The gradient consisted of 100% A for 0.5 min with a ramp up curve 6–100% B from 0.5 to 10 min.

The column eluent was introduced directly into the G2-S mass spectrometer by electrospray. Mass spectrometry was performed on a quadrupole-time-of-flight mass spectrometer operating in either negative or positive electrospray ionization. Positive mode had a capillary voltage of 3.00 kV and a sampling cone voltage of 30 V. Negative mode had a capillary voltage of 2.75 kV and had a sampling cone voltage of 20 V. The desolvation gas flow was set to 600 L/hour and the desolvation temperature was set to 500 °C. The cone gas flow was 25 L/hour and the source temperature was 100 °C. The data was acquired in the sensitivity MS mode with a scan time of 0.300 s and an interscan time of 0.014 s. Accurate mass was maintained by infusing Leucine Enkephalin (556.2771 *m*/*z*) in 50% aqueous acetonitrile (1.0 ng/mL) at a rate of 10 µL/min via the Lockspray interface every 10 s. Data was acquired in Centroid mode with a 50.0 to 1200.0 *m*/*z* mass range for TOF MS scanning.

Differentially expressed peaks between FLT-Sham and G-Sham were curated using a cut-off at a moderate *t*-test of *p* < 0.05. Following the same selection criteria, differentially expressed peaks were determined between FLT-Surgery and G-Surgery. The differentially expressed mass spectroscopy peaks were annotated using CEU Mass Mediator database (www.ceumass.eps.uspceu.es) and the molecules were screened based on the following guideline [51]: (a) ppm error<1; (b) chemical formula comprised of the adducts: +H, -H, +Na, +K, +NH4, -Cl; (c) annotation by Human Metabolome Database (HMD); and (d) chemical type of one of the following categories: (i) endogenous mammalian; (ii) drugs; (iii) toxicant; and (iv) reagents. Thereby, we identified the differentially expressed metabolites (DEMs).

### 2.9 Bioinformatics

Gene and metabolite knowledge integration was carried out at the network level. The DEGs and DEMs were uploaded to Ingenuity Pathways Analysis (IPA) platform (QIAGEN Inc., Valencia, CA) to curate the significantly enriched biological networks. The canonical networks were down selected based on the criteria set at the hypergeometric threshold of *p* < 0.05 and the total number of differentially expressed molecules enriching the network > 5. Here, the differentially expressed molecules were defined as the combination of the DEGs and DEMs. Similarly, the networks linked to diseases and non-canonical biological functions were curated upon the hypergeometric threshold *p* < 0.05 and the total number of differentially expressed molecules enriching the network > 50. Furthermore, a statistical approach provided by IPA was used to predict the regulation status of networks. Defined as z-score, this heuristic calculation takes into account the number of molecules enriching a particular network and their expression levels [52]. The networks showing z-score > 0 and < 0 were defined as activated and inhibited networks, respectively. The associations between the differentially perturbed canonical and non-canonical network were screened at the hypergeometric *t*-test *p* < 0.001.

### 2.10 Statistics

Statistical analyses were performed using GraphPad® version 5.0 software (GraphPad Software Inc., La Jolla, CA) and in house analytical pipelines developed in RStudio 0.99.902 (RStudio, Inc., USA).

The IPA platform (QIAGEN Inc., Valencia, CA) version 01–19-02 was used to mine the significantly enriched biological networks.

## 3 Results

### 3.1 Bone microarchitecture

Zero hardware failures were observed in FLT-Surgery group as none of the scaffolds was broken throughout the study. In the G-Surgery, one out of five scaffolds (20%) exhibited rupture during the study (Fig. 2A).

The microstructural analysis indicated no signs of bone bridging (RUST Score: 4 ± 0) for FLT-Surgery or G-Surgery mice (Fig. 2B), which would be expected with a critical sized SBD without additional therapeutic intervention, such as treatment with BMP-2. Table 1 shows the µCT data for microstructural parameters analyzed in the callus region between FLT-Surgery and G-Surgery mice. Although limited by sample size, spaceflight resulted in a 55% increase in trabecular separation (Tb.Sp; *p* = 0.07) and a 54% decrease in trabecular connectivity (Conn.D; *p* = 0.08). No differences were observed in total callus volume (TV) or in the percent of mineralized callus (BV/TV). Of note, the femur in which the scaffold broke (Fig. 2A) was excluded from these µCT analyses.

**Table 1:** Bone morphometric parameters for the segmental femoral defect measured by µCT in Ground and spaceflight surgery mice (G-Surgery vs. FLT-Surgery) after 24–28 days of space travel. *P*-values are noted; #0.1 ≤ p ≤ 0.05; not specific or ns 0.1 ≤ p. TV: tissue volume; BV: bone volume; Conn.D: connectivity density; SMI: structure model index; TMD: tissue mineral density; BS: bone surface; Tb.N: trabecular number; Tb.Th: trabecular thickness; Tb.Sp: trabecular separation.

	G-Surgery	FLT-Surgery	p-value
TV (mm^3^)	11.7 ± 9.3	15.7 ± 7.9	ns
BV (mm^3^)	1.9 ± 1.2	2.2 ± 0.6	ns
BV/TV (%)	17.8 ± 1.2	14.01 ± 0.6	ns
Conn.D (1/mm^3^)	185 ± 112	85 ± 34	p = 0.08 #
SMI	1.9 ± 0.1	1.7 ± 0.2	ns
TMD (1/cm^3^)	3.7 ± 0.4	3.9 ± 0.1	ns
BS (mm^2^)	74.3 ± 52.9	83.7 ± 18.0	ns
BS/BV (1/mm)	39.4 ± 7.4	38.1 ± 4.4	ns
Tb.N (1/mm)	5.0 ± 1.2	3.9 ± 0.8	ns
Tb.Th (mm)	0.08 ± 0.02	0.08 ± 0.01	ns
Tb.Sp. (mm)	0.22 ± 0.05	0.34 ± 0.08	p = 0.07 #

### 3.2 Transcriptomic assay results

The RNA qualities of bone tissues from both spaceflight and ground cohorts were comparable. The average RIN value was 2.1 (%CV = 0.33) and the DV_200_ value was 19.96 (%CV = 0.72). The Principal Component Analysis (PCA) of the global transcriptomic landscape demonstrated separation between the spaceflight and ground bone specimens in both Sham (Fig. S2A) and Surgery (Fig. S2B) groups. PC1 axis explained 34.7% and 23.3% of cumulative variance in the whole transcriptomic landscape of the Sham and Surgery group, respectively. The PC2 axis explained 16.4% and 21.8% of cumulative variance in the whole transcriptomic landscape of the Sham and Surgery group, respectively. However, no clear separation was observed in two-dimensional PCA figure when all Sham and Surgery cohorts across spaceflight and ground groups were included. Differential gene expression analysis found 452 DEGs between FLT-Sham and G-Sham, which included 366 upregulated and 86 down regulated genes in spaceflight versus ground specimens. There were 619 DEGs between FLT-Surgery and G-Surgery, which included 127 upregulated and 492 down regulated genes in spaceflight versus ground specimens. Interestingly, there were only 19 DEGs common between the Sham and Surgery groups across spaceflight and ground (Fig. S3A). Tables S2 and S3 list the DEGs enriching individual non-canonical and canonical networks, respectively.

### 3.3 Metabolomic assay results

Like the transcriptomic landscape, PCA of global metabolomics revealed considerable separation between spaceflight and ground bone specimens in the Sham (Fig. S2C) and Surgery (Fig. S2D) groups. To note, one of the four FLT-Surgery samples was clustered with G-Surgery group (Fig. S2D); however, such anomaly was not prominently repeated in other PCA plots. The spaceflight and ground specimens were separated along PC1 axis that explained 52.76% and 53.20% of cumulative variance in the Sham and Surgery group, respectively. The PC2 axis explained 26.50% and 34.65% of cumulative variance of the Sham and Surgery group, respectively. The differential metabolite expression analysis found 1,587 DEMs between FLT-Sham and G-Sham, which included 601 upregulated and 986 down regulated metabolites in spaceflight versus ground specimens. There were 1,520 DEMs between FLT-Surgery and G-Surgery, which included 703 upregulated and 817 down regulated metabolites in spaceflight versus ground specimens. One third of all DEMs or 808 DEMs emerged common between the Sham and Surgery groups across spaceflight and ground (Fig. S3B).

### 3.4 Gene-metabolite networks

**Fig. 3:**
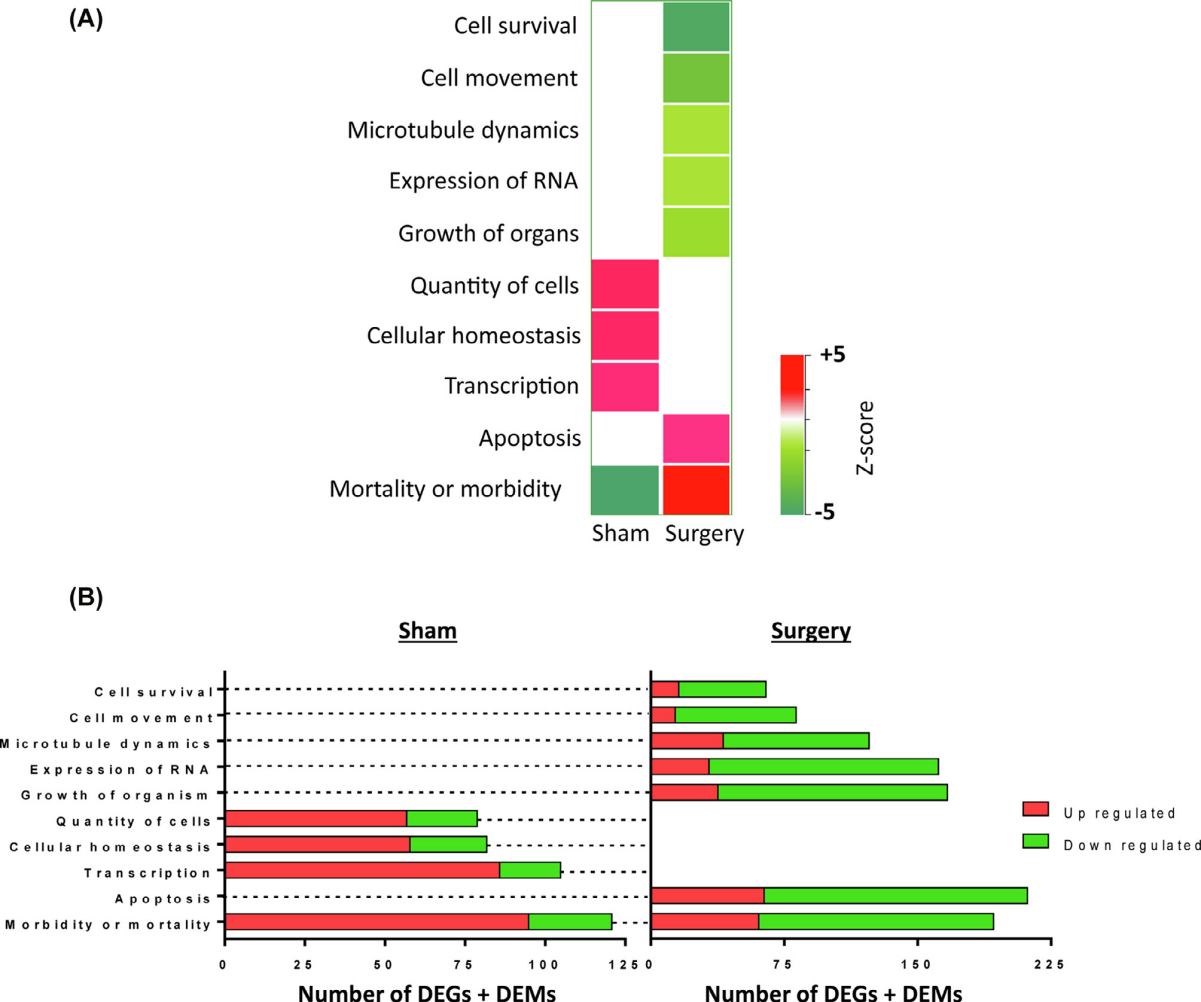
The biological functions. (A) Hierarchical clustering of the biological functions arranged according to their z-scores. In either Sham or Surgery groups, the activated and inhibited functions in the FLT-mice have z-scores greater than +2 or lower than −2, respectively than the G-mice. There are 4 and 7 biological functions significantly perturbed in Sham and Surgery mice, respectively. The function linked to morbidity is the only common function between Sham and Surgery, and it switches its regulation status between FLT-Sham and FLT-Surgery. Red and green represent activated and inhibited network in spaceflight, respectively (code seen bottom right). (B) Bar chart showing the distribution of upregulated and down regulated molecules in individual biological functions mentioned in Fig. 3A. By definition, the upregulated genes show higher genomic regulations in FLT-mice than G-mice; and vice versa. The horizontal axis represents the number of molecules enriching individual biological functions. The total number of molecules is defined as the sum of all differentially expressed genes (DEGs) and differentially expressed metabolites (DEMs). (For interpretation of the references to color in this figure legend, the reader is referred to the web version of this article.)

Significantly enriched diseases and non-canonical networks linked to Sham and Surgery groups are presented in Fig. 3A. Here the first column and second column are represented by the z scores associated with the FLT-Sham vs. G-Sham group and FLT-Surgery vs. G-Surgery group, respectively.

The bar graphs in Fig. 3B depict the total number of DEGs and DEMs enriching individual networks. The up-regulated DEGs and DEMs in Sham and Surgery groups are defined as those molecules that depicted higher regulation in spaceflight specimens compared to ground specimens, and vice versa. The Sham group enlisted four significantly perturbed non-canonical networks that included three activated networks and one inhibited network in the FLT-Sham group compared to the G-Sham group. The networks linked to transcription, cellular homeostasis, and quantity of cells were activated, and the network linked to morbidity was inhibited in FLT-Sham specimens compared to G-Sham specimens. However, the regulation status of the morbidity network switched in the Surgery cohort, as it emerged activated in FLT-Surgery mice in comparison to G-Surgery mice. The morbidity network in Sham and Surgery mice was enriched by 121 (104 DEGs and 17 DEMs) and 193 (143 DEGs and 50 DEMs) molecules, respectively with only 8 molecules common between the Sham and Surgery groups. To note, the morbidity network in the Sham group was primarily enriched by transcripts that were upregulated in FLT-Sham (95 upregulated and 26 downregulated transcripts in FLT-Sham in comparison to G-Sham; Fig. 3B). Contrastingly, the morbidity network in the Surgery group was primarily enriched by transcripts that were down regulated in FLT-Surgery (61 upregulated and 132 downregulated in FLT-Surgery in comparison to G-Surgery; Fig. 3B).

**Fig. 4:**
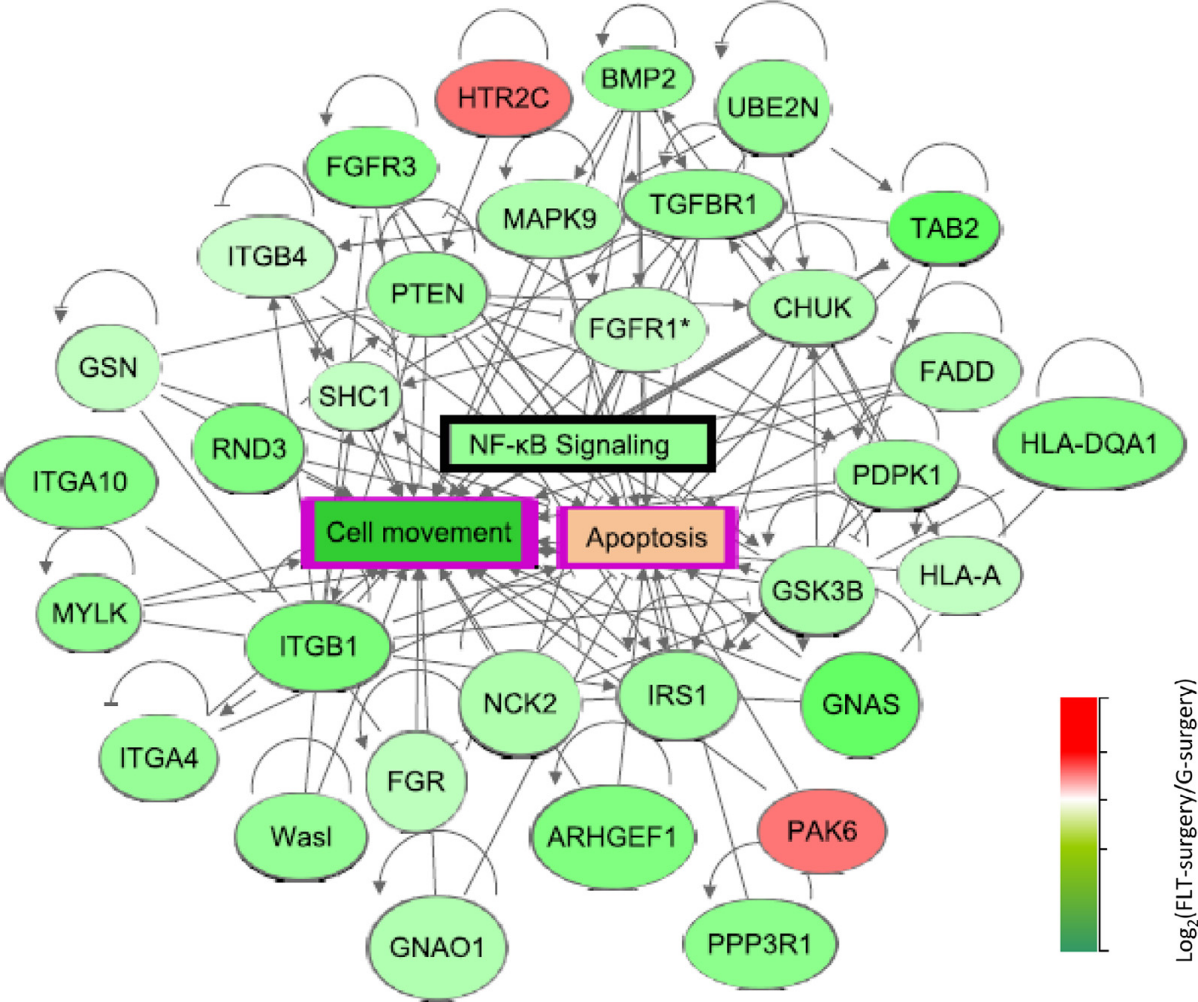
The canonical gene network linked to NFκB signaling in surgery mice. The oval and rectangular nodes represent genes and biological functions, respectively. The canonical signal, namely the NFκB network is inside a black-bordered rectangular box. Two associated networks, namely cell movement and apoptosis are contained within purple-bordered rectangular boxes. The oval and rectangular nodes are colored based on their regulation status. The genes upregulated in FLT-Surgery in comparison to G-Surgery are in red oval nodes; and the genes down regulated in FLT-Surgery in comparison to G-Surgery are in green oval nodes. The color code is at the bottom right. The edges connecting nodes represent their relationships. The arrow heads, blunt heads, and open end edges represent activating, inhibiting, and associative relationships, respectively. (For interpretation of the references to color in this figure legend, the reader is referred to the web version of this article.)

The gene-metabolite network linked to an apoptosis signal was significantly activated in the FLT-Surgery group compared to the G-Surgery group (Fig. 4). This network was enriched by 146 DEGs and 66 DEMs; and rather expectedly, the apoptosis network shared 105 and 82 differentially expressed molecules with the morbidity network and the cell survival network, respectively. Significantly inhibited in FLT-surgery mice, the cell survival network was enriched by 74 DEGs and 49 DEMs. In addition to the cell survival network, there were four networks inhibited in FLT-Surgery mice in comparison to G-Surgery mice, namely the networks linked to cell movement, microtubule dynamics, expression of RNA, and organism growth, respectively. Ranked by z-score, the gene-metabolite network linked to cell movement was one of the most inhibited networks in FLT-Surgery compared to G-Surgery, which was enriched by 116 DEGs and 51 DEMs. Table S2 lists the DEGs and DEMs enriching individual non-canonical networks.

**Fig. 5:**
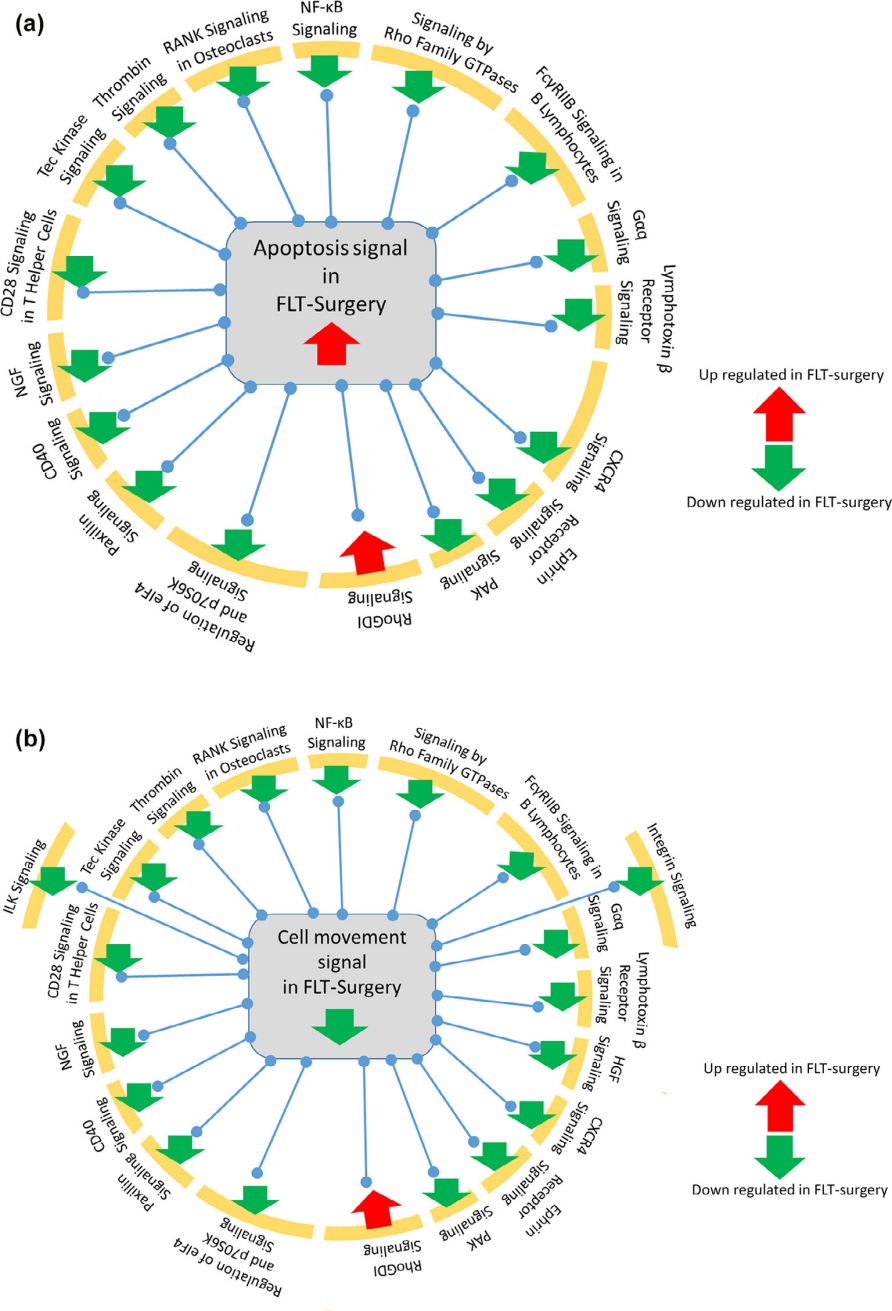
The interdependence between the biological functions and canonical networks in surgery mice. The focus was on two primary biological functions, namely apoptosis and cellular migration with surgery. The regulation status of these biological functions and canonical networks are defined by z-score. The activated and inhibited functions or networks in the spaceflight mice have z-scores greater than + 2 or lower than −2, respectively. The activation and inhibition status are represented as downward green arrows or an upward red arrow, respectively. The biological functions are at the center; while in the periphery, arranged are those canonical networks that have significant overlap with the respective biological function (hypergeometric *t*-test p < 0.05). The regulation status (activated vs. inhibited in FLT-Surgery) is noted by the arrows juxtaposing each network. (A) Apoptosis in FLT-Surgery. Apoptosis signal is activated in FLT-Surgery. There are 17 canonical networks driving the pro-apoptotic condition in FLT-Surgery. Except one activated network, namely RhoGDI signal, the remainder are inhibited in FLT-Surgery. (B) Cell movement in FLT-Surgery. This signal is inhibited in FLT-Surgery. In addition to the 18 canonical networks that are concurrently driving the pro-apoptotic and anti-migration status in FLT-Surgery, there are two canonical networks significantly associated with inhibited cell movement in the spaceflight callus, namely ILK signaling and integrin signaling. They are placed at the outer periphery and both are suppressed in FLT-Surgery. (For interpretation of the references to color in this figure legend, the reader is referred to the web version of this article.)

None of the canonical networks were significantly altered between FLT-Sham and G-Sham groups; while 25 canonical networks were significantly perturbed in FLT-Surgery versus G-Surgery groups. Among them, the Prostanoid Biosynthesis signal was exclusively enriched by DEMs and the remaining 24 canonical networks were exclusively enriched by DEGs. Table 2 lists these networks with brief descriptions, and Table S3 lists all candidate DEGs and DEMs enriching individual canonical networks. Rho GDP-dissociation inhibitor (RhoGDI) signal was the only activated canonical network in the FLT-Surgery versus G-Surgery groups. Ranked by z-score, the NFκB signal (Fig. 4) emerged as the most inhibited canonical network and this network was enriched by 10 DEGs. Fig. 5 depicted the panel of canonical networks that were significantly associated with the apoptosis (Fig. 5A) and cell movement (Fig. 5B) signals, respectively. The hypergeometric *t*-test identified 17 canonical networks that were significantly linked to the apoptosis network in the FLT-Surgery (Fig. 5A). Likewise, 20 canonical networks were significantly linked to the cell movement network in the FLT-Surgery (Fig. 5B). Several of the key gene candidates of these networks were validated using real-time PCR (Table 3).

**Table 2:** The list of canonical pathways significantly regulated in FLT-Surgery in comparison to G-Surgery. The pathways are sorted by the ascending order of z-score. The cut-off for significance of these pathways are –log(p-value) ≥ 1.3 and z-score > |2|. Pathways with z-score ≥ 2 and ≤ -2 are considered activated and inhibited pathways, respectively. N or Number of molecules (DEGs + DEMs) enriching individual networks are listed, and subsets of these molecules overlapping with apoptosis and cell movement are listed in parenthesis.

Canonical Pathways	-log(p-value)	z-score	N (apoptosis/cell movement)
NF-κB Signaling	1.74	−3.16	10 (9/9)
RANK Signaling in Osteoclasts	2.37	−2.83	8 (8/7)
Thrombin Signaling	2.19	−2.83	12 (10/10)
PKCθ Signaling in T Lymphocytes	1.63	−2.83	9 (5/5)
iCOS-iCOSL Signaling in T Helper Cells	4.19	−2.71	12 (8/8)
Tec Kinase Signaling	2.93	−2.71	12 (12/12)
CD28 Signaling in T Helper Cells	2.73	−2.65	10 (7/7)
NGF Signaling	1.44	−2.65	7 (7/7)
Role of NFAT in Regulation of the Immune Response	3.47	−2.50	14 (10/8)
Signaling by Rho Family GTPases	2.37	−2.50	14 (12/12)
FcγRIIB Signaling in B Lymphocytes	2.24	−2.45	7 (6/6)
HGF Signaling	1.51	−2.45	7 (7/6)
Gαq Signaling	1.71	−2.33	9 (8/7)
Integrin Signaling	4.41	−2.32	17 (13/15)
Lymphotoxin β Receptor Signaling	1.55	−2.24	5 (5/5)
CD40 Signaling	1.3	−2.24	5 (5/5)
ILK Signaling	4.47	−2.138	15 (11/13)
Paxillin Signaling	3.07	−2.12	10 (8/9)
Regulation of eIF4 and p70S6K Signaling	1.66	−2.12	9 (7/7)
CXCR4 Signaling	1.56	−2.12	9 (8/8)
Ephrin Receptor Signaling	1.49	−2.12	9 (7/9)
PAK Signaling	4.08	−2.11	11 (9/10)
NER Pathway	1.37	−2.00	6 (1/1)
RhoGDI Signaling	1.52	2.12	9 (7/7)

**Table 3:** The list of genes validated by qPCR. The fold change value (i.e. the ratio of gene regulations in FLT-Surgery and G-Surgery are Log2 transformed and presented in array values’ descending order). The genes enriching for the NFκB signal are underlined.

Gene ID	Gene name	Log_2_(Fold change); Fold change = (FLT-Surgery/ G-Surgery)		Relevance to present study
		Array	qPCR	
GAB3	Growth Factor Receptor Bound Protein 2-Associated Protein 3	1.93	1.13	Check points of growth factors and cytokines
OSTM1	Osteoclastogenesis Associated Transmembrane Protein 1	1.92	1.55	Key regulator of bone resorption [85]
FGFR1	Fibroblast Growth Factor Receptor 1	1.14	1.05	Consistently overexpressed during facture healing to potentially promote callus remodelling [86]
CHUK	Component of Inhibitor of NFκB Kinase Complex	−1.77	−1.73	Upstream regulator of NFκB signal
MAPK9	Mitogen-Activated Protein Kinase 9	−1.78	−0.30	Critical player in bone formation [87]
GSK3B	Glycogen Synthase Kinase 3 Beta	−1.90	−0.33	Promoter of delayed bone maturation via selective gene deletion [88]
IRS1	Insulin Receptor Substrate 1	−2.14	−0.58	Promoter osteoblast proliferation and bone elongation [89]
PDPK1	3-Phosphoinositide Dependent Protein Kinase 1	−2.17	−1.30	Encoding protein that activates NFκB signal [90]
TGFBR1	Transforming Growth Factor Beta Receptor 1	−2.31	−1.73	Regulator of wound regeneration as associated via point mutation [91]
BMP2	Bone morphogenetic protein 2	−2.37	−0.73	Encoding key protein for bone regeneration and repair
PPP3R1	Protein Phosphatase 3 Regulatory Subunit B, Alpha	−2.50	−0.31	Regulator of calcium binding process, a key mechanism of bone fracture healing
RND3	Rho Family GTPase 3	−2.74	−0.81	Key regulator of cell proliferation and apoptosis [92]
MYBPC1	Myosin Binding Protein C1	−3.39	−0.71	Encoding myosin associated protein
MYL3	Myosin Light Chain 3	−3.65	−3.23	Encoding a key skeletal muscle protein
MYOZ2	Myozenin 2	−4.50	−0.77	Encoding another sarcomere skeletal muscle

## 4 Discussion

### 4.1 RR4, a unique condition to promote musculoskeletal atrophy

Astronauts returning from space have experienced 1–1.6% per month loss of lower limb bone mineral density [5] and a 15%-20% per year reduction in the bending and compressive strength of bones [53]. Considering that fewer than 600 humans have travelled to space, and most of them stayed for less than three weeks in low-earth orbit, little is known about the impact of long-term spaceflight on the musculoskeletal system. It remains elusive whether the rapid and intense rate of bone loss in spaceflight will persist over a multiple-year mission or if it will diminish over time to reach an equilibrium state; and rather importantly, how this compromised musculoskeletal health will affect bone injuries and repair processes. To address these questions, the RR4 project supported the first space mission to study a male mouse cohort that underwent a SBD surgery in their femoral midshaft. SBD healing in a mouse femur represents a challenging, yet practical bone fracture model.

Conventional musculoskeletal injury models on ground have major limitations in simulating the human paradigm. The reason is the following- the rodents ambulate, bearing weight on their injured limbs immediately after the anesthetic wears off; whereas humans with similar limb injuries have limited to no weight bearing for a significant period of time, of up to 6 months [28]. Hence, the acute mechanical loading due to pre-mature ambulation in rodents, potentially improves fracture healing; contrastingly, a prolonged unloading is likely to impair human bone healing efficacy. In potential agreement, we observed that at least one of the five scaffolds (20%) broke in the G-Surgery mice; on the other hand, no scaffold breakage was observed in FLT-Surgery mice, likely attributed to the weightlessness condition in spaceflight that allowed prolonged unloading of the rodents’ weight bearing limbs [28]. While the differential acute and premature applications of weight bearing on the femoral SBD in spaceflight vs. on ground could be the cause of the scaffold breakage, no affirmative conclusion is drawn due to the small sample size. In this context, it is important to note that our past observation using the same model showed that the scaffold structure and hardware (hollow needle) was able to tolerate the vibration and hypergravity associated with launch, and the healing dynamics were most likely not influenced by the spaceflight launch [28]. Taking all of these factors in account, examining fracture healing in spaceflight arguably has some critical advantages over conventional Earth-based model.

The RR4 project presented an opportunity to probe the potential SBD healing over 24–28 days of onboard space mission. By estimation, the 28 days of rodent life is equivalent to more than two years of human lifetime [54] and nearly 4% of rodent life; hence, RR4 mice spent a much longer fraction of their life in space than humans recorded to date. Previous studies examining the femur of RR4 sham mice reported significant reductions in trabecular bone volume fraction (BV/TV, 25% decrease) and trabecular number (Tb.N, 4.9% decrease), associated with a significant increase in trabecular separation (Tb.Sp, 6.9% increase) in spaceflight (FLT-Sham) compared to ground (G-Sham) femurs [4]. Expanding on this report, the present study probed the impact of the RR4 spaceflight mission on the healing of a femoral SBD. The defect size inflicted in the RR4 mice is considered a critical sized defect, which by definition, is not expected to be healed without therapeutic intervention [28]. Indeed our µCT data demonstrated no sign of bridging in either spaceflight (FLT-Surgery) and ground (G-Surgery) mice. Rather subtle signs, such as increased trabecular spacing and decreased trabecular connectivity in the spaceflight callus suggested an escalated risk of bone non-union that might correlate with poor bone quality and lower mechanical integrity in spaceflight.

### 4.2 Gene-metabolite networks linked to spaceflight induced bone loss

Spaceflight emerged as the primary factor to explain the genomic and metabolomic perturbations in both Sham and Surgery groups. Reduced femoral bone mass in FLT-Sham mice co-occurred with the activated molecular signal linked to cellular homeostasis. Balancing the extracellular stress and intracellular responses, the cellular homeostasis signal plays a major role in aiding cell survival [55]. In agreement, the morbidity signal was inhibited in FLT-Sham mice. This cumulative picture of the Sham network analysis possibly suggests an underlying molecular program that acted against further femoral bone loss in spaceflight 24–28 days post-launch. A recent study has drawn a similar conclusion investigating hindlimb unloading (HLU) as a musculoskeletal disuse model on ground [56]. In their study, 14-days long HLU triggered an early onset of apoptosis in the femoral osteocyte cells, which reached a plateau between 5 and 15 days since the inception of HLU model. This putative arresting of apoptosis signal was concurrent with an escalation of trabecular bone resorption [56]. Interestingly, at least two other studies demonstrate the role of unloading-induced osteocyte apoptosis in the regulation of bone resorption [57], [58], which is consistent with the current studies showing the upregulation of the apoptosis signal in FLT-Surgery mice.

In the Sham mice, there was a potential dissociation at the transcriptomic level between muscle and bone in responding to the spaceflight induced stress. Our recent analysis of the quadriceps muscle found a significant suppression of bioenergetics that was orchestrated with immune deficiency and metabolic dysfunction in FLT-Sham mice in comparison to G-Sham mice after 24–28 days space travel [9]. However, the apoptosis gene networks and bioenergetics networks linked to genes and metabolites were unperturbed in the FLT-Sham femurs compared to G-Sham femurs. Therefore, we speculate that the muscle tissue possibly showed a delayed adaptability to the stress in comparison to the bone tissues, which is a typical characteristic differences between the healing trajectories of muscle and bone, respectively [59].

### 4.3 SBD, a dominating factor over spaceflight impacts

The molecular landscape of FLT-Surgery callus depicted a very different scenario from FLT-Sham femur. The molecular shifts of the sham femur towards the adaptation of the spaceflight induced stress were overwhelmed by the new surge of molecular perturbations triggered by SBD in spaceflight. In FLT-Surgery callus, the active morbidity signal was synchronized with the upregulated apoptosis signal and suppressed cell migration network.

Theoretically, the longitudinal dynamics of fracture healing is a multi-phasic biomechanical process [60] that begins with an inflammatory phase, when cytokines and growth factors begin migrating from the neighboring cells to induce the osteogenic and angiogenic activities at the fracture site [61]. The selective recruitment of extracellular matrix (ECM) components stimulates the activation of mesenchymal stem cells (MSCs), chondrocytes, osteoblasts, fibroblasts, and endothelial cells. These cumulative efforts ultimately reach a milestone, at which time bone bridging across the fracture site begins. Expectedly, the critical sized bone defects in the RR4 mice failed to reach this milestone, as we found no sign of significant callus formation in the SBD of either the FLT-Surgery or G-Surgery groups. The molecular landscape in FLT-Surgery was particularly unfavorable for healing; the inhibition of the cell migration signal may have further impacted the failure of the spaceflight callus to recruit the key agents to activate MSCs and other cellular components essential for callus formation.

### 4.4 Potential roles of gene-metabolite networks impeding healing in spaceflight

Migration of ECM to the callus is facilitated by the cross-collaboration among integrin, microtubule, and actin-myosin filaments [62], and all of these cytoskeletal elements were adversely affected in spaceflight. In FLT-Surgery, the inhibited integrin-NFκB interaction, a key modulator of cell migration [63], potentially operated via an inhibited intermediator, namely the Tec kinase signal [64]. Furthermore, a compromised actin-myosin filament bundle in the FLT-Surgery callus was speculated, since a number of genes encoding myosin related proteins, such as MYL3, MYLK, MYB, and MYCPB were inhibited in FLT-Surgery compared to G-Surgery. The ‘master modulator’ of actin signal, namely Rho-family GTPase [65], which is known to reorganize the actin-myosin complex in non-muscular cells [66], became inhibited in FLT-Surgery callus. In harmony, BMP-2 gene that encodes an activator protein [67] and Rho GDP-dissociation inhibitor (RhoGDI) gene that encodes an inhibitor protein [68] of Rho family GTPases emerged downregulated and activated, respectively in FLT-Surgery compared to G-Surgery. Of interest, BMP is a major chemoattractant of ECM [10]; interestingly, BMP-7 gene was upregulated in FLT-Sham callus in comparison to G-Sham callus, but BMP-2 gene was downregulated in FLT-Surgery callus in comparison to G-Surgery callus. To note, both BMP-2 and -7 proteins are important in bone healing and they both trigger osteogenic and osteoclastic activities [69], although their degree of performances vary [70].

The migration of the ECM components during the early bone fracture-healing phase typically coincides with the infiltration of T helper (T_h_) cells and thrombin to trigger inflammation and coagulation. Expression of NF-κB in T_h_ cells has been attributed to the T_h_ cell-induced immunological and apoptotic activities [71]. Operation of T_h_ cells is contingent upon the co-stimulatory activities of CD28 and CD40 pathways [72]. We speculate that the suppression of both CD28 and CD40 networks in the FLT-Surgery callus could be attributed to the inactivation of the T_h_ cells or reduced load of T_h_ cells due to disrupted trafficking, or a combination of the two. A cluster of inhibited networks linked to cell migration potentially inform the impaired recruitment in callus; however, it is beyond the scope of RR4 study design to directly confirm the reduced load of T_h_ cells in the callus.

**Fig. 6:**
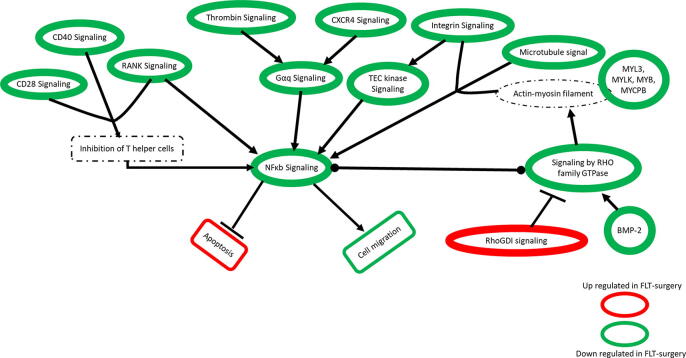
The pro-apoptotic and anti-cell migratory cluster of networks in FLT-Surgery. These networks put forward synergistic efforts converging towards the inhibited NFκB signal. The oval and circular nodes represent individual networks or differentially expressed genes, respectively. The rectangle with solid border represent the biological functions of interest. The molecular signals with broken borders are those, whose activation status could not be evaluated directly; yet there were many indirect evidences suggesting their potential engagement in the given cluster of networks. The color of the node’s solid border represents its regulation status in FLT-Surgery. Activated and inhibited signals in FLT-Surgery are represented by green and red colored borders, respectively. The edges represent the relationships between two nodes. The nodes with arrowheads, blunt heads, or circular ends represent the activating, inhibiting, or associative relationship between the two connecting nodes, respectively. (For interpretation of the references to color in this figure legend, the reader is referred to the web version of this article.)

Furthermore, SBD in spaceflight fostered a comprehensive inhibition of a procoagulant-antiapoptotic axis composed by the Thrombin signal and Gαq signal converging to the NFκB signal (Fig. 6) [73], [74]. Other key procoagulant and angiogenic signals such as CXCR4 that depends on NFκB transcriptional activities [75] emerged inhibited in FLT-Surgery callus in comparison to G-Surgery callus. Together, the NF-κB signal emerged as a critical hub network in the spaceflight callus, where most of the other significantly perturbed signals converged to exploit the pro-apoptotic and anti-migration scope of inhibited NF-κB [76], [77]. Moreover, the receptor activator of nuclear factor κB (RANK) gene, which became inhibited in FLT-Surgery encodes a well-established regulator of bone resorption [78], [79].

### 4.5 Limitations and conclusions

The limitations of our study are primarily due to the inherent constraints associated with spaceflight as well as limitations of the SBD healing model. For instance, there are certain technical barriers and limited footprints allotted to conduct biological research on the ISS. Standard immunological tests to directly monitor the apoptosis or live cell migration are still not feasible to carry out on the ISS. Due to the small sample size, the histological study was precluded, and we relied on the integrative multi-omics approaches to support our claims. Extraction of high quality RNA from bone is always a challenge; and present protocol involved a freeze–thaw cycle that potentially further compromised the RNA quality extracted from bone. Hence, we were limited to run the cDNA array using RNA samples with sub-optimal quality. To mitigate the risk of generating false results, we administered strict cut-offs in the downstream analysis and validated results using multiple assays. We were refrained from determining any biomarkers associated with spaceflight induced musculoskeletal atrophy; rather this study was focused on informing about how a cluster of molecules and networks could respond to the stress compounding spaceflight and bone fracture. Gene perturbations are typically associated with early response to any extracellular stimuli; while the metabolites are generally the final derivative of biological functions. Therefore, the integrative gene and metabolite approach can inform time-dependent consequences of stress. The RR4 mission exclusively used male mice; hence, we could not illustrate sex-based differences in regulating the musculoskeletal health and bone repair [80], [81].

With unlimited resources, many different or additional experimental design elements would have been added to these studies including the use of multiple fracture healing models rather than single SBD model, as a wide range of information can be obtained from different models. Additionally, multiple time points would have been examined including earlier and later time points. Indeed, earlier time points would have provided additional important information related to the molecular factors that govern whether a fracture will heal or not (possible biomarkers). Here an approximately 4 week time point was examined. The rationale was that for a critical sized defect model with use of thrombopoietin as a therapeutic for bone healing, complete bridging is not observed until 6–8 weeks post-surgery, whereas with use of BMP-2 complete bone bridging is observed approximately 2 weeks post-surgery [82], [83] and remodeling phases are typically complete by about 12 weeks post-surgery. It was therefore hoped that a 4 week time point would capture the active callus formation phase of bone healing. Further, both male and female mice as well as different ages of mice would be used if not for inherent spaceflight constraints. Moreover, surgeries would be completed in spaceflight as compared to on Earth so that the entire healing process would occur in space.

With regard to the selection of the SBD healing model, because an important goal of ongoing investigations is to determine what therapeutics could be used to improve bone healing in humans during longer-term space stays, a SBD healing model which allowed for local delivery of drugs to the fracture site was used. This model was further selected because of its many clinically relevant features (e.g. use of the same type of collagen sponge used to deliver BMP-2 to human patients, use of hollow rods, implantation of the collagen sponge at the time of surgical stabilization), the reproducibility of the fracture pattern, and the reduction in soft tissue injury compared to the crush injury experienced to the quadriceps when using the closed Einhorn fracture model [25]. However, there are many disadvantages to the use of this model, compared to a more traditional closed model such as the Einhorn fracture model. An important limitation in this control study, where drugs such as BMP-2 were not administered, is that as observed in Fig. 2, limited callus formation was observed which decreased the amount of callus available for use in our omics analyses. The use of the scaffold adds another confounding variable to our omics analyses as does the use of the collagen sponge.

Limitations aside, our genome-to-phenome integrative approach suggested spaceflight as a highly unfavorable environment for fracture healing. The recruitment of regeneration-critical cellular and molecular agents at the wound site was potentially interrupted in spaceflight. The inhibited NF-κB signal potentially facilitated dual adverse functions, namely promoting apoptosis and impeding cell migration [79], [84]. This node could be an ideal target for precision medicine approaches, but a delicate balance is required to achieve maximum benefit, since NF-κB is a ubiquitous regulator of transcription.

## CRediT authorship contribution statement

**Nabarun Chakraborty:** Conceptualization, Formal analysis, Project administration, Writing - original draft, Writing - review & editing. **Ariane Zamarioli:** Methodology, Investigation, Resources, Writing - review & editing. **Aarti Gautam:** Methodology, Validation, Resources, Investigation. **Ross Campbell:** Formal analysis. **Stephen Mendenhall:** Methodology. **Paul J. Childress:** Methodology, Investigation. **George Dimitrov:** Methodology. **Bintu Sowe:** Methodology. **Aamir Tucker:** Methodology. **Liming Zhao:** Methodology, Investigation. **Rasha Hammamieh:** Conceptualization, Supervision, Project administration, Funding acquisition, Writing - review & editing. **Melissa A. Kacena:** Conceptualization, Supervision, Project administration, Funding acquisition, Writing - review & editing.

## Declaration of Competing Interest

The authors declare that they have no known competing financial interests or personal relationships that could have appeared to influence the work reported in this paper.
